# Children’s mental health problems and their relation to parental stress in foster mothers and fathers

**DOI:** 10.1186/s13034-017-0180-5

**Published:** 2017-09-06

**Authors:** Arnold Lohaus, Sabrina Chodura, Christine Möller, Tabea Symanzik, Daniela Ehrenberg, Ann-Katrin Job, Vanessa Reindl, Kerstin Konrad, Nina Heinrichs

**Affiliations:** 1 Faculty of Psychology and Sports Sciences, University of Bielefeld, P.O. Box 10 01 31, 33501 Bielefeld, Germany; 2 University of Braunschweig, Institute of Psychology, Humboldtstr. 33, 38106 Brunswick, Germany; 3 Department for Child and Adolescent Psychiatry, University Hospital Aachen, Neuenhoferweg 21, 52074 Aachen, Germany

**Keywords:** Parental stress, Foster families, Mental health problems, Internalizing behavior, Externalizing behavior

## Abstract

**Background:**

This study focuses on children living in foster families with a history of maltreatment or neglect. These children often show adverse mental health outcomes reflected in increased externalizing and internalizing problems. It is expected that these adverse outcomes are associated with increased parental stress levels experienced by foster mothers as well as foster fathers.

**Methods:**

The study sample included 79 children living in foster families and 140 children living in biological families as comparison group. The age of the children ranged from 2 to 7 years. Mental health problems were assessed with the Child Behavior Checklist, while parenting stress was measured with a parenting stress questionnaire including subscales on the amount of experienced stress and the amount of perceived support. The Child Behavior Checklist assessments were based mainly on maternal reports, while the parental stress assessments were based on maternal as well as paternal reports.

**Results:**

As expected the results showed increased externalizing and internalizing scores for the foster children accompanied by increased parental stress experiences in the foster family sample (however only in the maternal, but not in the paternal stress reports). The stress differences between the foster and biological family groups disappeared, when the children’s mental health problem scores were included as covariates. Moreover, especially the externalizing scores were strong predictors of parental stress in both, the groups of foster and biological parents. The amount of perceived social support was associated with reduced parental stress, but only in the group of biological fathers.

**Conclusion:**

The emergence of parental stress in biological as well as foster parents is closely related to child characteristics (mainly externalizing child problems). Possible implications for the reduction of parental stress are discussed as a consequence of the present results.

## Background

When children are allocated to a foster family, they often look back at a history of maltreatment experiences during the time when they lived in their biological families. Childhood maltreatment is associated with a range of emotional and behavioral problems. Maltreated children show significantly more externalizing and internalizing symptoms, more discipline problems in school and more symptoms of depression than children without such experiences [1, 2]. As a consequence, foster parents are confronted with increased demands, which may induce parental stress as an aversive psychological reaction to the demands of being a parent [3].

Several previous studies showed associations between parental stress and child mental health problems in non-foster parents. For example, a study by Mesman and Koot [4] found significant relations between parental stress and the extent of externalizing and internalizing symptoms in children aged 10–11. The association to parental stress was closer for externalizing in comparison to internalizing symptoms. This is also underlined by studies addressing attention deficit hyperactivity disorder (ADHD) problems. As a meta-analysis by Theule et al. [5] showed, parents of children with ADHD reported more parenting stress than parents of nonclinical controls. Moreover, the severity of the ADHD symptoms was associated with parenting stress, especially in combination with conduct problems (e.g., oppositional behavior). Associations to parental stress were also found for children with developmental delays [6], for children with autism spectrum disorders [7], for children with sleep disturbances [8] and for children with chronic diseases [9]. In general, mental as well as somatic problems are typically associated with increased demands for parents, which are often reflected in increased parental stress perceptions.

Parenting might in some respects be even more demanding for foster parents. On the one hand, foster children may exhibit increased emotional and behavioral problems as a result of previous maltreatment experiences [10]. On the other hand, children and foster parents are unfamiliar with each other when the children enter their new families. This means that emotional ties and familiar behavior patterns may emerge over time, but are not available from the beginning. This is an important difference to many other challenging child conditions, because, in general, children with mental or somatic problems live in their familiar environment. Thus, the perceived parental stress may even be increased in foster parents, because they are confronted with an unfamiliar child with potential mental health problems.

Increased perceived parental stress may be associated with reduced parenting capacities. As a study by Farmer, Lipscombe and Moyers [11] for foster caregivers of adolescents showed, conduct problems, hyperactivity, and violent behavior shown by the adolescents increased caregivers’ strain. Increased caregivers’ strain, on the other hand, was associated with significantly higher disruption rates (which indicates increased mutual interaction problems). Thus, increased parental stress may reduce the quality of the parent–child-interaction and thus may contribute to an increase of child problems. The authors also found that the perceived strain was reduced when caregivers received help from friends and local professionals, which underlines the role of social support in reducing stress experiences.

Although relations between challenging child characteristics and parental stress have been addressed in previous studies, most of these studies were related to non-foster contexts, while empirical studies focusing on stress perceptions of foster parents—especially in children—are scarce. Previous studies used parenting stress as outcome measure in parent training for foster parents [12], included parenting stress as control variable in studying parenting practices of foster parents [13] or were related to specific subgroups, e.g. parenting stress in adolescent mothers in foster care [14]. A study by Jiménez et al. [15] is related to parental stress in kinship foster families, which, however, are only in part comparable to the situation of non-kinship foster families addressed in the current study. One of the few studies which are directly related to parental stress in foster parents is a study of Nadeem et al. [16] with repeated assessments of foster children’s mental health problems and their foster parent’s parental stress. This study showed associations between children’s problems and parental stress and, moreover, changes of parenting stress across repeated assessments (at 2 months, 12 months and 5 years post-placement), but included no comparison group. The current study is directly related to comparing the parenting stress of non-kinship foster parents with biological parents. The focus is on stress perceptions of both foster mothers and foster fathers. Although fathers are also involved in parenting, the majority of previous studies focused on mothers only, because they are typically the primary reference persons for children. To our knowledge, this is the first study that does not only include foster mothers, but also foster fathers.

It is hypothesized that the perceived level of stress in foster parents and also the extent of children’s mental health problems are increased in comparison to control parents living exclusively with their biological children (Hypothesis 1) and that the differences between the stress levels of foster and biological parents are expected to disappear by controlling for the extent of the children’s mental health problems (Hypothesis 2). In addition, it is assumed that the level of perceived parental stress is closely related to the extent of the children’s mental health problems (in foster as well as biological families) and that social support perceived by the parents decreases the level of perceived parental stress (Hypothesis 3). Because little is known about the relation between children’s mental health problems and parenting stress in preschool and elementary school age children (especially in foster families), the focus of the current investigation is on young children aged 2–7 years.

## Methods

### Sample

The data of the current investigation are obtained from the GROW&TREAT foster family study, funded by the Federal Ministry of Education and Research. The total sample of the GROW&TREAT project consisted of 94 foster children and 157 children living in their biological families. Only non-kinship foster care families were included in the foster sample. Most foster families were recruited from youth welfare offices at three regions in Germany (up to 200 km around Aachen, Bielefeld, and Braunschweig). The biological parents were recruited from the same regions with postings or at parents’ evenings in nursery and elementary schools. If the foster or the biological families had more than one child in the target age, they were asked to select one child as the target child (based on a random choice). However, in some cases, more than one child of a foster or biological family was selected as target child. This was the case for 15 foster and 17 biological families. To avoid dependencies within the data set, these families were excluded from the analyses reported in this study. Thus, the final sample of this study included 79 children living in foster families and 140 children living in biological families. In the final sample, the age of the children varied between 2 and 7 years [*M* = 3.49 (*SD* = 1.32) for the foster children and *M* = 4.40 (*SD* = 1.41) for the biological children]. A *t* test indicated a significant age difference between the two groups *t* = 4.65, *df* = 217, *p* < .001, but there were no significant differences with regard to the children’s sex. Because of the significant age difference, all statistical analyses in the “Results” section included age as covariate. The foster children lived in their foster families since *M* = 17.72 months (*SD* = 8.61). The most important sample characteristics are provided in Table 1. Participation of the foster families required the permission of the foster parents, the youth welfare office, and the biological mother or the legal guardian. The procedure and assessments were approved by an independent ethics committee. The foster as well as the biological families received 30 Euros as incentive for participation at the assessments included in this study. For more information on the GROW&TREAT project and on the complete assessments see http://www.grow-and-treat.de.

**Table 1:** Sample characteristics of the recruited samples (children living in foster and biological families)

	Foster families	Biological families	Statistical test
Children’s age	*M* = 3.49 (*SD* = 1.32)	*M* = 4.40 (*SD* = 1.41)	*t* = 4.65, *df* = 217, *p* < .001
Children’s sex	39 female, 40 male	76 female, 64 male	*χ* ^2^ = .49, *p* = .484
Age of the mother	*M* = 40.54 (*SD* = 6.81)	*M* = 35.38 (*SD* = 5.40)	*t* = 6.17, *df* = 217, *p* < .001
Age of the father	*M* = 44.01 (*SD* = 6.73)	*M* = 38.62 (SD = 6.01)	*t* = 5.96, *df* = 209, *p* < .001
Time in foster family (in months)	*M* = 17.72 (*SD* = 8.61)		

### Measures

*Parental stress* was assessed by the Parental Stress Questionnaire [17], which includes a 17-item subscale (Parental Distress Subscale) related to the degree of experienced parental stress (item example: “I struggle a lot with my child”). Moreover, there are subscales related to perceived social support in general (item example: “I have people in my surrounding who might watch my child”) and to perceived social support by the partner (item example: “My partner supports me in the education of our child”). The latter subscale had to be completed only if a partner was available. A forth subscale of the Parental Stress Questionnaire (Role Restriction) was not provided in the context of this study. All items had to be assessed on a 4-point Likert scale (0 = “strongly disagree”, 1 = “disagree”, 2 = “agree” and 3 = “strongly agree”). For further analyses sum scores were calculated for the three subscales (separately for mothers and fathers). It should be noted that there are two versions of the parental distress subscale for parents of preschool and school children. Thus, the parents were asked to complete the school version if their child already attended a school. Five items of the parental distress subscale are reformulated in the school version to meet the specific demands of parents of older children. As a consequence, the scale values of the two versions were z-standardized (across the foster and non foster groups, but separately for the preschool and school versions and separately for mothers and fathers) to adjust the values to comparable ranges. The calculations in the “Results” section are based on these z values. The mothers as well as the fathers in foster and biological families were asked to complete the subscales. Data for the parental stress subscale were available from 72 foster mothers. For the general social support and the partner support subscale, data were provided from 76 respectively 70 foster mothers. The respective sample sizes for foster fathers were 66, 69, and 68. In the case of biological families, the sample sizes were 130, 131, and 121 (mothers) and 111, 116, and 114 (fathers). The internal consistencies of the subscales are provided in Table 2. Across samples, the correspondence between the assessments of fathers and mothers was *r* = .38, *p* < .001 for the parental distress subscale. The respective values for the general social support and the partner support subscale were *r* = .51, *p* < .001, and *r* = .35, *p* < .001.

**Table 2:** Internal consistencies for the questionnaire scales included in this study

	Foster families	Biological families	Total
Maternal distress—preschool	.88	.89	.89
Maternal distress—school	.93	.90	.92
Mothers’ perceived social support—general	.78	.80	.79
Mothers’ perceived social support—partner	.80	.83	.82
Paternal distress—preschool	.88	.89	.88
Paternal distress—school	.95	.85	.87
Fathers’ perceived social support—general	.79	.68	.73
Fathers’ perceived social support—partner	.45	.76	.71
CBCL—total score—age range 2–4	.95	.97	.96
CBCL—externalizing problems—age range 2–4	.89	.89	.90
CBCL—internalizing problems—age range 2–4	.91	.81	.90
CBCL—total score—age range 5–7	.94	.89	.91
CBCL—externalizing problems—age range 5–7	.89	.86	.89
CBCL—internalizing problems—age range 5–7	.74	.76	.75

*Psychological symptoms* of the children were assessed using German versions of the Child Behavior Checklist (CBCL). For children in an age range from 2 to 4 years, the CBCL 1½–5 was used [18], while the CBCL 4–18 was used in the age range from 5 to 7 [19]. The CBCL reports were typically only provided by the mothers (in 81.2% of the cases), while 7.7% of the assessments were provided by fathers and 11.1% completed the CBCL jointly. In line with the manual’s instructions, a total problem score was calculated as well as scores for the broad-band scales for internalizing and externalizing syndromes. As previously described for parenting stress, the scale values for the CBCL 1½–5 and the CBCL 4–18 were z-standardized separately to adjust the values of the different versions to comparable ranges. The internal consistencies are provided in Table 2. The externalizing and internalizing scale values correlated *r* = .65, *p* < .001 across samples.

### Statistical analyses

The comparisons of the perceived level of stress and the extent of children’s mental health problems between the groups of foster and biological parents were based on analyses of variance (Hypothesis 1). In the case of externalizing and internalizing problems, multivariate analysis of variance was used to account for the substantial correlations between these dependent variables. Hypothesis 2 was tested by including the extent of the children’s mental health problems as a covariate in the analysis of variance addressing parenting stress differences between foster and biological parents. Hierarchical regression analyses were used to analyze the contribution of children’s mental health problems and perceived social support (in general and by the partner) to parental stress (Hypothesis 3). Because of missing data in some assessments, the sample sizes may vary across the analyses, as can be seen by the degrees of freedom or by the sample sizes reported in the Tables.

## Results

### Children’s mental health problems and parental stress in foster vs. biological families

To address Hypothesis 1, a univariate analysis of variance was calculated with family type (foster vs. biological) as independent variable and the total CBCL score as dependent variable. The age of the children was included as covariate. The results indicated a significant difference for the total CBCL score (*F*_1,209_ = 29.30, *p* < .001, *η* ^2^ = .123) with increased values in the foster children (see Table 3). There was no additional age effect (*F*_1,209_ = .93, *p* = .337, *η* ^2^ = .004).

**Table 3:** CBCL and parental stress scores in foster and biological families (based on z values)

	Foster families			Biological families		
	Mean *z* value	*SD*	*n*	Mean *z* value	*SD*	*n*
CBCL total score	.47	1.20	78	−.24	.75	134
CBCL externalizing score	.47	1.19	78	−.24	.78	134
CBCL internalizing score	.33	1.24	78	−.19	.74	134
Maternal parental distress (scale)	.28	.98	72	−.16	.98	130
Paternal parental distress (scale)	.06	1.00	66	−.04	1.00	111

A multivariate analysis of variance with the internalizing and externalizing CBCL scales as dependent variables and age as covariate underlines the result for the total score. There is a significant multivariate difference (*F*_2,208_ = 13.57, *p* < .001, *η* ^2^ = .115). Moreover, the univariate analyses indicated significant differences for both the internalizing (*F*_1,209_ = 14.27, *p* < .001, *η* ^2^ = .070) and the externalizing scale (*F*_1,209_ = 26.30, *p* < .001, *η* ^2^ = .112). Again, there was no significant age effect. In both cases, the Child Behavior Checklist scores are increased for the group of foster children (see Table 3).

Analyses of variance were calculated for the parental distress subscale as dependent variable for mothers and fathers separately. The age of the children was again included as covariate. The results for the mothers indicated significant effects for the parental distress subscale (*F*_1,199_ = 10.04, *p* = .002, *η* ^2^ = .048) indicating increased parental stress in the group of foster mothers (see Table 3). The results for the fathers revealed no significant differences for the parenting distress subscale. Moreover, there was no effect of age as covariate.

To summarize, the parents noticed increased mental health problems in foster care children and especially the foster mothers perceived in addition increased parental stress. To address the question, whether the parental stress differences between foster and biological mothers are due to increased mental health problems in foster children, the analyses of variance, which were calculated to address Hypothesis 1, were recalculated including the total CBCL scores as covariate (in addition to age). Confirming Hypothesis 2, the analysis of variance did not indicate any parental stress difference between foster and biological mothers. These calculations were repeated using the externalizing and the internalizing problem scores as covariates (in separate analyses). Again, the differences between the two groups disappeared after including the child behavior problem scores in the analyses.

### Relation between children’s mental health problems and parental stress

Table 4 shows the Pearson correlations between the CBCL scores and the parental stress indicators. As can be seen, there are substantial correlations between both variable sets. As can be expected, the stress experienced by the parents was increased if they noticed mental health problems in their children (in foster as well as in biological parents). In general, the correlations seem to be increased for externalizing in comparison to internalizing problems.

**Table 4:** Correlations between CBCL and parental stress scores in foster and biological families and in the total sample

	CBCL total score	CBCL externalizing score	CBCL internalizing score
Total sample			
Maternal parental distress (scale)	.52*** (n = 198)	.57*** (n = 198)	.34*** (n = 198)
Paternal parental distress (scale)	.33*** (n = 172)	.38*** (n = 172)	.16* (n = 172)
Foster families			
Maternal parental distress (scale)	.43*** (n = 69)	.48*** (n = 69)	.30* (n = 69)
Paternal parental distress (scale)	.34** (n = 63)	.36*** (n = 63)	.17 (n = 63)
Biological families			
Maternal parental distress (scale)	.55*** (n = 124)	.61*** (n = 124)	.33*** (n = 124)
Paternal parental distress (scale)	.33*** (n = 106)	.40*** (n = 106)	.13 (n = 106)

* *p* < .05, ** *p* < .01, *** *p* < .001

To analyse the relative contribution of externalizing and internalizing problems to parental stress, hierarchical regression analyses were calculated. In the first step of the analyses, externalizing and internalizing scores were included as predictors, in the second step the general social support and social support by the partner were added as predictors to be able to analyse the role of perceived social support for parental stress (Hypothesis 3). The results are shown in Table 5 for maternal and in Table 6 for paternal stress.

**Table 5:** Prediction of maternal stress in foster and biological families by CBCL scores (externalizing and internalizing) and perceived social support

	*B*	*SE B*	*β*	*p*	Δ*r* ^*2*^	Significance of Δ*r* ^*2*^
Foster families—maternal stress						
Step 1—predictors					.387	<.001
CBCL externalizing score	.808	.114	.640	<.001		
CBCL internalizing score	−.045	.127	−.043	.726		
Step 2—predictors					.014	.274
CBCL externalizing score	.776	.118	.615	<.001		
CBCL internalizing score	−.052	.127	−.037	.681		
Social support—general	.100	.070	.107	.153		
Social support—partner	−.077	.077	−.078	.319		
Biological families—maternal stress						
Step 1—predictors					.198	.001
CBCL externalizing score	.354	.115	.440	.006		
CBCL internalizing score	.005	.106	.007	.961		
Step 2—predictors					.012	.628
CBCL externalizing score	.337	.119	.419	.006		
CBCL internalizing score	.002	.119	.002	.988		
Social support—general	−.047	.116	−.048	.682		
Social support—partner	−.096	.109	−.103	.380		

n = 67 in foster families and n = 117 in biological families

**Table 6:** Prediction of paternal stress in foster and biological families by CBCL scores (externalizing and internalizing) and perceived social support

	*B*	*SE B*	*β*	*p*	Δ*r* ^2^	Significance of Δ*r* ^2^
Foster families—paternal stress						
Step 1—predictors					.139	.009
CBCL externalizing score	.364	.129	.428	.007		
CBCL internalizing score	−.079	.120	−.100	.514		
Step 2—predictors					.046	.188
CBCL externalizing score	.331	.129	.390	.013		
CBCL internalizing score	−.082	.118	−.104	.492		
Social support—general	−.037	.115	−.038	.748		
Social support—partner	−.295	.163	−.212	.075		
Biological families—paternal stress						
Step 1—predictors					.167	<.001
CBCL externalizing score	.600	.141	.469	<.001		
CBCL internalizing score	−.186	.159	−.129	.244		
Step 2—predictors					.122	<.001
CBCL externalizing score	.491	.135	.384	<.001		
CBCL internalizing score	−.228	.151	−.158	.132		
Social support—general	.086	.090	.083	.342		
Social support—partner	−.333	.080	−.369	<.001		

n = 65 in foster families and n = 105 in biological families

As the results for step 1 show, externalizing problems were the most important predictor for maternal as well as paternal stress (in both foster and biological families). Internalizing problems did not additionally contribute to the explanation of variance in parental stress. If the social support variables were included, there was no significant increase in the explanation of maternal stress. This result was comparable for foster fathers, but there was a significant stress decrease in biological fathers if they experienced increased support by their partners.

To summarize, there are substantial relations between parental stress and children’s externalizing CBCL scores, and a contribution of perceived social support could only be identified for biological fathers.

## Discussion

According to Abidin [20] there are many possible sources of parental stress. Relevant stressors are related to work, environment, marital relationship, daily hassles, life events, parent characteristics, and child characteristics. This study focused mainly on emotional and behavioral problems as specific child characteristics, which may be associated with increased parental stress. As the results for Hypothesis 1 show, there is evidence that parents of foster children are confronted with increased levels of child behavioral and emotional problems. It is well known from previous studies that children in foster care usually show higher levels of behavioral and emotional problems compared to children living with their biological parents [21, 22]. While many previous studies focused on elementary school aged children and on adolescents, the present study shows that these results may also be extended to preschool age children.

At the same time, especially foster mothers reported increased parental stress. However, the differences between the stress levels of foster and biological mothers disappeared when the children’s mental health problems were included as covariates in the analyses. Thus, our findings support the assumption that large contributions to the emergence of stress in foster families may be related to child characteristics (especially behavioral and emotional problems), at least in foster mothers. It is, however, unclear how this relation emerges, because it is also possible that parents perceiving increased strain are less effective in parenting and caregiving which may also result in a close relation between children’s problems and parental stress. Thus, it is possible that there are bidirectional relations between parental stress and child characteristics. Independent of the direction, it might—as a consequence—be helpful to support foster parents in caring for children with such problems in order to reduce parental stress.

It is interesting to note that the association of parental stress to child characteristics is lower for internalizing than externalizing problems [23]. The reason may be that internalizing problems are hardly identified by external observations—an effect well known from studies on cross-informant discrepancies [24–26]. Similar results were shown in a previous study by Mesman and Koot [4] who found closer associations between externalizing problems and parental stress than between internalizing problems and parental stress. According to Mesman and Koot [4] externalizing behavior problems are not only more observable for parents, but they also require more attention. As a consequence, this relation is shown not only in foster parents, but also in biological parents, although foster parents may be confronted with even more child behavior problems. The decreased observability may also explain the insignificant correlations between internalizing problems and paternal stress in Table 4 (in foster families as well as in biological families). In many families, the mothers spend more time with their children than the fathers, which may lead to an increased chance for mothers to perceive internalizing problems. Although the regression analyses underline that externalizing problems are generally more closely associated with parental stress than internalizing problems, it should be noted that this study is restricted to young children and that externalizing behavior may be more salient for parents at this age. Thus, it is unclear whether the contribution of externalizing problems to parental stress in comparison to internalizing problems will change, when the children grow older.

Although a significant contribution of perceived social support to the reduction of parental stress was assumed, this could only be found for biological fathers. In this case, perceived social support by the partner contributed to decreased parental stress. It should, however, be noted that social support may not only lead to decreased parental stress, but that increased parental stress may also lead to increased social support to cope with a demanding situation. Thus, there may be a mutual influence in both directions, which may explain the absence of substantial relations between both variable sets in most analyses. The situation may also be different in foster parents who receive, at least in some communities in Germany, additional professional social support by foster family support groups or supervisory meetings, etc.

It is additionally interesting to note that there were no sex differences regarding the results of this study. It is well known from the literature that externalizing problems are typically more often shown by boys and internalizing problems by girls. These differences emerge, however, more clearly during early adolescence, but seem to be less prominent during childhood [27, 28]. This could explain the absence of sex influences in the included age range.

## Conclusions

In sum, our results indicate that there is a close association between children’s mental health problems and parental stress and that this is true for biological as well as for foster families. Assuming a bidirectional relationship between parental stress and child behavior problems, there might be at least three possible implications of the current study results for the reduction of parental stress: (1) interventions with a focus on the parent (training of coping strategies, strengthening parental resources etc.), (2) interventions with a focus on the child (interventions treating children’s mental health problems to indirectly influence parental stress), and (3) interventions related to parent–child interactions (to improve the mutual adjustment of parents and their children). Although all three approaches may be promising in reducing parental stress and improving a child’s well-being, it may depend on the specific situation of the family and of the target child, which strategy is most appropriate. An intervention example is the keeping foster parents trained and supported (KEEP) approach [29], which equips foster parents with strategies to manage externalizing behavior problems. The program has been shown to be effective in reducing children’s problem behavior and in reducing parental stress levels.

According to Chamberlain et al. [30] the number of child problem behaviors is linearly related to the risk of placement disruption during subsequent years. The perceived parental stress may be an important mediator variable in this relationship. On the other hand, placement disruptions increase the risk for child problem behaviors. To avoid vicious circles it is important to provide appropriate interventions at early stages of the development of problem behavior. Foster parents need support because of their duties and challenges on many different levels. Therefore they have to be well prepared and trained and the social services should provide easy accessible structures of help to support foster parents. Generally, a close collaboration between social services and clinical child and adolescent psychological services should be strengthened. Further research is needed to identify specific stressors in the context of parental stress and to develop appropriate prevention and intervention support programs.

A possible weakness of this study may be seen in the recruitment of the samples, because there might be self-selection effects, which may reduce the representativeness of the samples. It should, however, be noted that the mean CBCL-T values of the control children were 49.71 for externalizing and 50.31 for internalizing problems which is very close to the T = 50 value expected for the total population. However, the T value calculations had to be based on American norm data, because there are no specific German norm data for the CBCL-version used for younger children. Based on the available norm data, there is no indication that the children from the biological families represent a specific population with improved mental health. It should also be noted that the foster families may be representative for a relatively highly educated sample. This, however, represents the typical living conditions of foster children after a placement in a new family in Germany, because most foster families are well educated with a specific interest in improving the well-being of children.

A specific strength of the current study is that it compares parental stress and children’s mental health problems in comparably large samples of foster and biological parents. Previous studies were typically related to the role of child characteristics for parental stress in non-foster samples, while few studies were directly related to parental stress in foster family samples comparing the parental stress of foster and biological parents at early stages of their children’s life. Moreover, there are very few studies focusing not only on maternal stress, but also on paternal stress. To our knowledge, there was no previous study including paternal stress in a foster family sample. Thus, the study at hand has broadened the knowledge about the relations between children’s behavior problems and parental stress especially in foster families.

## Abbreviations

ADHD: attention deficit hyperactivity disorder
CBCL: Child Behavior Checklist
KEEP: keeping foster parents trained and supported
